# Superpixel-Based Temporally Aligned Representation for Video-Based Person Re-Identification †

**DOI:** 10.3390/s19183861

**Published:** 2019-09-06

**Authors:** Changxin Gao, Jin Wang, Leyuan Liu, Jin-Gang Yu, Nong Sang

**Affiliations:** 1 Key Laboratory of Ministry of Education for Image Processing and Intelligent Control, School of Artificial Intelligence and Automation, Huazhong University of Science and Technology, Wuhan 430074, China; cgao@hust.edu.cn (C.G.); jinw@hust.edu.cn (J.W.); 2 National Engineering Research Center for E-Learning, Central China Normal University, Wuhan 430079, China; lyliu@mail.ccnu.edu.cn; 3 School of Automation Science and Engineering, South China University of Technology, Guangzhou 510640, China; jingangyu@scut.edu.cn

**Keywords:** person re-identification, superpixel, temporally aligned pooling, walking cycle

## Abstract

Most existing person re-identification methods focus on matching still person images across non-overlapping camera views. Despite their excellent performance in some circumstances, these methods still suffer from occlusion and the changes of pose, viewpoint or lighting. Video-based re-id is a natural way to overcome these problems, by exploiting space–time information from videos. One of the most challenging problems in video-based person re-identification is temporal alignment, in addition to spatial alignment. To address the problem, we propose an effective superpixel-based temporally aligned representation for video-based person re-identification, which represents a video sequence only using one walking cycle. Particularly, we first build a candidate set of walking cycles by extracting motion information at superpixel level, which is more robust than that at the pixel level. Then, from the candidate set, we propose an effective criterion to select the walking cycle most matching the intrinsic periodicity property of walking persons. Finally, we propose a temporally aligned pooling scheme to describe the video data in the selected walking cycle. In addition, to characterize the individual still images in the cycle, we propose a superpixel-based representation to improve spatial alignment. Extensive experimental results on three public datasets demonstrate the effectiveness of the proposed method compared with the state-of-the-art approaches.

## 1. Introduction

Person re-identification (re-id), as an important technique to automatically match a specific person in a non-overlapping multi-camera network, has been widely used in many applications, such as surveillance [1,2], forensic search [3], and multimedia analysis [4]. It is a challenging problem because of large variations in a person’s appearance caused by illumination, pose or viewpoint changes, as well as occlusion. Two fundamental problems in person re-identification intensively studied in the literature [3,5,6,7] are feature representation [8,9,10,11,12,13,14] and metric learning [15,16,17,18,19,20,21,22,23,24,25], and this work is mainly concerned with the former.

Existing works on feature representation mostly rely on still person images across non-overlapping camera views. These works can further be divided into two groups according to the number of images they use, namely, single-shot re-id and multiple-shot re-id. Single-shot re-id methods use one single image to model the person, which are evidently limited in that they do not make full use of the information. Because, in real-world applications (like surveillance), there is usually a sequence of images available for a person in each camera view. As a consequence, the single-shot methods often suffer from some practical challenging factors, like occlusion, and pose, viewpoint or lighting changes. Multiple-shot re-id methods [26,27,28,29] can alleviate these issues to some extent by utilizing more images in their feature representations. However, the multiple-shot re-id methods still have two typical limitations: (1) they treat a video sequence as an unordered set of images, where the temporal information is totally lost; (2) they may be computationally costly since appearance features need to be calculated on a large number of frames.

To tackle the limitations mentioned above, some authors recently advocate video-based re-id, which can usually achieve better performance by exploiting the abundant space–time information in feature representation. Compared to still-image-based re-id, where only spatial alignment needs to be considered since the primary challenge in feature representation is to achieve robustness to viewpoint changes, one fundamental but challenging problem in video-based re-id is temporal alignment. Considering its significance, temporal alignment has been studied in very recent literature [30,31], both using Flow Energy Profile (FEP) to align the video sequences temporally. The FEP extracts the motion information the optic flow field. Wang et al. extract fixed-length fragments around the local maxima/minima of FEP [30], and Zhang et al. use the frequency of dominant one in the discrete Fourier transform domain of FEP to extract a more stable walking cycle [31]. However, they still suffer from the following problems: (1) FEP, captured using optic flow, is based on individual pixels, and all the pixels of the lower body are considered. That means FEP is pixel level motion information, which is less accurate or robust due to the heavy noise caused by background clutter or occlusions. (2) These works represent the video using all the motion information [30,31], i.e., around all the local maxima/minima of FEP or all the walking cycles. However, this to some extent introduces redundancy and noise caused by cluttered background and occlusions, which is harmful to robust representations. Thus, we argue that person representation using only one walking cycle may be a more effective strategy to address the problem of redundancy and noise. However, the walking cycle should representative, i.e., with less redundancy and less noise. Although some deep learning-based methods have been proposed recently, they still depend on much more resources of computation, memory, training data, compared to the traditional methods. This makes the deep learning based method can not be used in some resource limited applications.

To this end, we present a superpixel-based Temporally Aligned Representation (STAR) method to address the temporal alignment problem for video-based person re-identification. More precisely, we first extract motion information from the input sequence by tracking the superpixel of the lowest portions of human, to build a candidate set of walking cycles. Second, on the assumption that there is a whole walking cycle in the video of each person, we select the “best” walking cycle to perform temporal alignment across videos. Finally, to extract the representation of the selected walking cycle of a video, we propose a superpixel-based representation for each single image, and a walking cycle based temporally aligned pooling method.

The preliminary version of portions of this paper has been published in [32]. Compared to [32], this manuscript (1) proposes a superpixel based representation for the still images, termed as SPLOMO, and compares it with original LOMO; (2) expands it from 5 pages to more than 17 pages; (3) expands or rewrites all the sections, and adds the “Related work” section; (4) in Experimental Results Section, adds some recent works in the comparison results, and adds the “Evaluation of Components and Parameters”, analyzing the results in more detail.

To sum up, the contributions of this paper are as follows:

(1) We propose a robust temporal alignment method for video-based person re-id, which is featured by the superpixel-based motion information extraction, the effective criterion for candidate walking cycles, and the use of only the best walking cycle to build the appearance representation.
(2) Superpixel-based support regions and person masks are introduced to still image representation, so as to improve spatial alignment and thereby alleviate the undesired effects of the background.
(3) The proposed method, performs favorably against the state-of-the-art methods, even deep learning-based ones.

The rest of this paper is organized as follows. Related work is reviewed in Section 2. We introduce the proposed STAR video representation in detail in Section 3. In Section 4, we present extensive experimental results, and we conclude this paper in Section 5.

## 2. Related Work

Person re-id has attracted much attention in recent years, and we first point the readers to some literature surveys on this topic [3,5,6,7,33]. Many methods focus on tackling this problem, which can be roughly divided into three categories, i.e., feature representation [8,34,35], metric learning [36,37,38,39] and deep learning [40,41,42,43,44,45,46,47,48]. Since this paper focuses on the video-based re-id task, this section only gives a review of the literature closely related to this work. Particularly, we first compare the difference between multiple-shot re-id and video-based re-id and point out the critical problem in video-based re-id, followed by reviewing the video-based re-id methods according to its main steps.

### 2.1. Multiple-Shot Re-Id vs. Video-Based Re-Id

In real video surveillance applications, the information for a person is recorded by a video or an image sequence, rather than a single image. Person re-id based on video is an effective way, due to abundant information in videos, such as underlying dynamic information. Some works have validated the superiority of video-based re-id methods [30,31,49,50,51].

The direct way to use the video information is the multiple-shot re-id methods [26,27,28,29,52]. Some state-of-the-art methods select key frames from the video sequence and then process them like the still images [40,52,53]. However, the multiple-shot methods ignore the dynamic information in the videos. Therefore, the video-based re-id methods are recently proposed [30,31,52,54,55,56,57,58]. To avoid confusion, we point out that the difference between the multiple-shot re-id and the video based re-id: the multiple-shot re-id utilizes the video as an unordered image set; while the video based re-id considers the video as an image sequence with the space–time information (i.e., motion information). As mentioned before, temporal information is the main difference between multiple-shot re-id and video-based re-id. However, as the image sequences of different persons are unsynchronized, it is not easy to build a robust representation which contains motion information. Therefore, the critical problem in the video-based re-identification is to synchronize the starting/ending frames of the image sequences of different persons according to the motion information, termed as temporal alignment. For instance, the fragments of complete walking cycles or gait periods can be selected from the video sequences, to build robust spatial–temporal representations.

### 2.2. Video-Based Re-Id Methods

According to its characteristics, we list three main steps in the video-based re-id methods: temporal alignment, spatial–temporal representation, and metric learning. Many works related to these steps have been proposed recently:

- Temporal alignment. Temporal alignment has been demonstrated to be able to lead to a robust video-based representation in the context of gait recognition [59]. Some recent works try to consider this problem in video-based re-id [30,31]. Inspired by motion energy in gait recognition [59], Wang et al. propose to use Flow Energy Profile (FEP) to describe the motion of the two legs, and employe its local maxima/minima to temporally aligned the video sequences [30]. To improve the robustness of temporal alignment, Liu et al. proposed to use the frequency of dominant one in the discrete Fourier transform domain of FEP [31].
  Although the methods [30,31] have demonstrated the effectiveness of FEP based temporal alignment, they still suffer from heavy noise due to cluttered background and occlusions, since they apply optic flow to extract the motion information based on all the pixels of the lower body including the background. Moreover, in real video surveillance applications, the video sequence of a person usually contains several walking cycles, which is redundant.
  To address the aforementioned problems, this paper proposes a superpixel based temporal alignment method, by first extracting the superpixels on lowest portions of human in the first frame, and then tracking them to obtain the curves of their horizontal displacements, finally selecting the “best” cycle in the curves. Note that only one cycle is used for person representation, to address the problem of redundancy and cluttered background and occlusions. Our work is partially inspired by two aforementioned video-based re-id approaches [30,31]. However, our proposed method essentially departs from these existing methods in the following three aspects:
  (1) We adopt a superpixel-based strategy to extract the walking cycles of walking person, i.e., by tracking the superpixels of the lowest portions of human (like feet, ankles, or legs near the ankles), instead of relying on the motion information of all the pixels of the lower body as in previous approaches [30,31], which is less accurate or robust due to the heavy noise caused by background clutter or occlusions.
  (2) We propose to use only the best walking cycle for temporal alignment, rather than using all the walking cycles. An effective criterion based on the intrinsic periodicity property of walking persons is proposed to select the best walking cycle from the motion information of all the superpixels. The motion information of a superpixel matches the human walking pattern, to some extent means the superpixel lies on person, and not be occluded.
  (3) Based on our temporal alignment, we introduce a novel temporally aligned pooling method to establish the final feature representation. More specifically, we take superpixels as the region supports to characterize the individual still images. Meanwhile, we then utilize person masks to enhance the robustness further.
- Spatial–temporal representation. The gait based feature is the reliable information for person re-id [59]. However, it suffers from occlusions and cluttered background. Most spatial–temporal representations for video-based re-id are devised by considering the videos as 3D volumes, inspired by the some existing works of action recognition, for instance, 3D-SIFT [60], extended SURF [61], HOG3D [62], local trinary patterns [63], motion boundary histograms (MBH) [64]. Recently, some spatio–temporal representation methods have been proposed for person re-id [30,31,57,65,66,67]. For instance, Wang et al. use HOG3D [30], Liu et al. aggregate a 3D low-level spatial–temporal descriptors into a single Fisher Vector (STFV3D) [31], and Liu et al. propose Fast Adaptive Spatio-Temporal 3D feature (FAST3D) for video-based re-id [57]. Being built upon 3D volumes, these appearance based spatial–temporal representations also consider the dynamic motion information to some extent. It is worth noting that, combining with temporal alignment, these representations are more robust.
  Considering that most 3D representations are extensions to some widely used 2D descriptors, this paper proposes a simple framework to build a 3D representation, by combining single image based representations and temporally aligned pooling. Another reason is that lots of successful single image based representations have been proposed for person re-id, which are person re-id specific, such as LOcal Maximal Occurrence representation (LOMO) [8], Gaussian Of Gaussian (GOG) [35], and so forth. To introduce these features to 3D video data representations, we propose the temporally aligned pooling to integrate all single image based features to form a spatial–temporal representation.
- Metric learning. Learning a reliable metric for video matching is another important factor for the video-based re-id [30,54,55,56,68,69]. Recently, some works have been proposed for video matching. For instance, Simonnet et al. introduce Dynamic Time Warping (DTW) distance to metric learning for the video-based re-id [54]; Wang et al. propose Discriminative Video fragments selection and Ranking (DVR) method for video matching [30]. Based on the observation that the inter-class distances with the video-based representation are much smaller than that with single image based representation, You et al. propose a top-push distance learning model (TDL) for the video-based re-id [56].
  If we extract a fixed-length representation for a video sequence, the metric learning methods for video-based re-id is same with that for single-image-based re-id. Thus, the single-image-based metric learning methods can be used directly, such as KISSME in [8,31]. In this paper, we obtain the fixed-length representations by temporally aligned pooling and use Cross-view Quadratic Discriminant Analysis (XQDA) [8], a well known single-image-based metric learning method, for video matching.

It is stressed that deep networks based frameworks do not follow the steps mentioned above, but train an end-to-end neural network architecture [28,52,53,58,70,71,72,73,74]. For instance, McLaughlin et al. proposed to represent the appearance of the video sequences by a convolutional neural network (CNN) model, and represent the temporal information by a recurrent layer [70]. Although they achieve good performance, the deep learning based methods are still limited to the resources of computation, memory, and training data.

## 3. Superpixel-Based Temporally Aligned Representation

**Figure 1:**
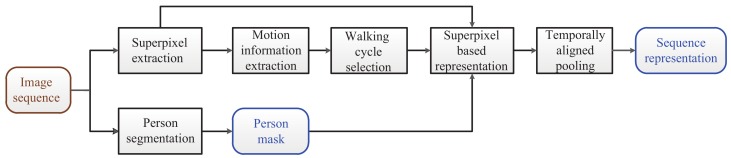
Framework of the proposed superpixel-based temporally aligned representation.

This paper focuses on appearance representation for video-based person re-id. In this section, we introduce the proposed superpixel-based temporally aligned representation by (1) extracting motion information based on superpixel tracking (Section 3.1), (2) selecting the “best” walking cycle using an unsupervised method (Section 3.2), and (3) constructing a 3D representation based on superpixel-based representation (Section 3.3) and temporally aligned pooling (Section 3.4). We depict the entire framework in Figure 1.

### 3.1. Motion Information Extraction

We propose a superpixel based motion information extraction, which ought to be more robust than pixel based methods, because (1) it is based on superpixels, dynamic information extraction is robust to noise caused by some individual pixels, while pixels based on methods like FEP suffer from this; (2) to alleviate the effect of occlusions and cluttered background, we utilize the local superpixel to extract motion information, and then select the “best” cycle in the curves of all the superpixels. Note that, although the superpixels may also be on the background, whose curves of motion information are quite easy to be distinguished from that on person, as discussed in Section 3.2 and Section 4.3.

**Figure 2:**
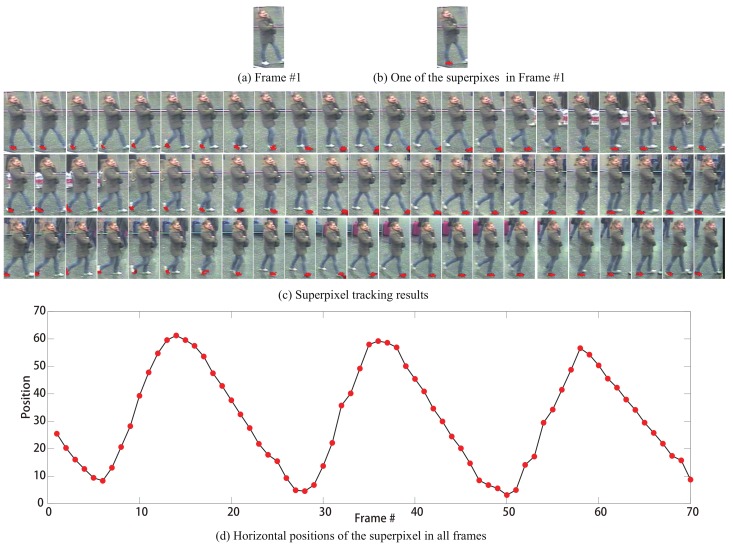
Motion information extraction based on superpixel tracking. (**a**) The image of frame #1; (**b**) One of the superpixels in frame #1, labeled in red color; (**c**) The superpixel tracking results in the images of frame #2 to #70, labeled in red color; (**d**) Horizontal positions (red dots) of the superpixel in all frames. The figure is best viewed in color.

Given a video sequence $V={\left\{I_{t}\right\}}_{t=1,\ldots ,T}$, with *T* frames, we extract the motion information, as illustrated in Figure 2. In our implementation, we only consider the motion information of the lowest portions of human, because of its amplitude of walking is more significant. Specifically, we first perform superpixel segmentation on the lowest portion of the first frame, using SLIC method [75]. *N* superpixels ${\left\{S_{1}^{j}\right\}}_{j=1,\ldots ,N}$, are obtained. Figure 2b shows an example, where the superpixel labeled in red is on the right foot of the person.

Then we track all the *N* superpixel to extract the motion information. For the *j*th superpixel $S_{1}^{j}$, we track it throughout the video sequence *V*, resulting in a set of superpixel ${\left\{ST_{t}^{j}\right\}}_{t=1,\ldots ,T}$ containing *T* elements, where $\left\{ST_{t}^{j}\right\}$ is from the *t*th frame. Note that, although we use a set to describe the superpixels, it is ordered, and the superpixel in the sets are in the same order as the frames in the corresponding video.

At frame *t*, we extract $N_{t}$ superpixels using SLIC, that is ${\left\{S_{t}^{k}\right\}}_{k=1,\ldots ,N_{t}}$. For simplicity, we obtain the tracking result $ST_{t}^{j}$, which is the best match in ${\left\{S_{t}^{k}\right\}}_{k=1,\ldots ,N_{t}}$ to the initial superpixel $S_{1}^{j}$, with the smallest distance:

(1) $$ST_{t}^{j}=S_{t}^{k^{*}}=\underset{k}{\arg\min}{\left(f(S_{1}^{j})-f(S_{t}^{k})\right)}^{2},$$

where f(x) denotes the representation of superpixel *x*. In this paper, we use the color feature, i.e., HSV histogram, to represent the superpixels. Figure 2c shows the superpixel tracking results corresponding to the initial superpixel in Figure 2b. We denote the horizontal positions ${\left\{L_{t}^{j}\right\}}_{t=1,\ldots ,T,j=1,\ldots ,N}$ of the centers of the superpixel set ${\left\{ST_{t}^{j}\right\}}_{t=1,\ldots ,T,j=1,\ldots ,N}$. The final motion information of the region corresponding to the *j*th superpixel $S_{1}^{j}$ can be described as ${\left\{L_{t}^{j}\right\}}_{t=1,\ldots ,T}$, as shown in Figure 2d. We can see that the superpixels along the entire video sequence is about the right foot. There is a high probability that it is a part of a person with somewhat semantic information, which makes the motion information extraction algorithm very robust.

**Figure 3:**
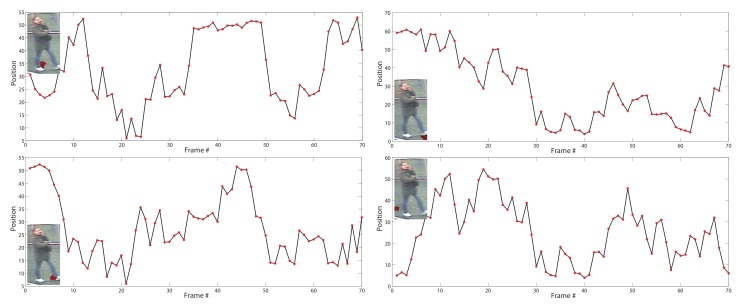
Motion information of four superpixels on the background, from the same sequence in Figure 2. The figure is best viewed in color.

It is worth noting that we track all the superpixel on the lowest portion of an image, without segmenting the lowest portions of human out of the background. The main reasons are two-fold: (1) foreground segmentation is not dependable since it is difficult due to noise, occlusions, and low-resolution (see the last row in Figure 9); (2) the motion information of the superpixels in the background actually is easy to eliminate, since the motion information of the superpixels from the foreground and from the background in the lowest portion of the frame is quite different. Figure 3 presents the motion information of some superpixels on the background. We can see that their motion information is quite different from that of the superpixels on person as shown in Figure 2, and do not match the intrinsic periodicity property of walking persons. This observation indicates that we can easily select “good” walking cycles from noise and redundancy motion information, as described in Section 3.2.

### 3.2. Walking Cycle Selection

**Figure 4:**
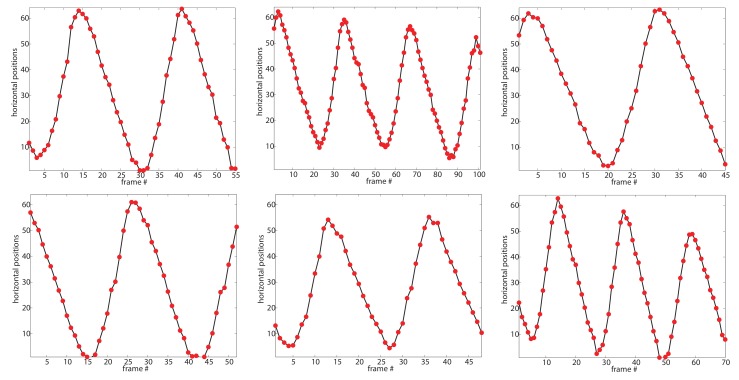
Illustration of motion information of feet with some examples on the iLIDS-VID dataset. We annotate the mean horizontal positions of the right foot of a person’s image sequence, and then we show the horizontal positions (red dots) with frame index.

To address the problem of abundance and noise caused by cluttered background and occlusion, we use only one walk cycle of frames to represent a video sequence. Therefore, after obtaining the redundant motion information (i.e., horizontal positions) ${\left\{L_{t}^{j}\right\}}_{j=1,\ldots ,N,t=1,\ldots ,T}$ of the *N* superpixels, we are trying to select the “best” walking cycle $\left(t_{start}^{*},t_{end}^{*}\right)$. The motion information of a superpixel of the lowest portions of human is described as its horizontal displacements with time as in Section 3.1. The fragments of the motion information can be considered as the candidate walking cycles. Then, a natural question is: what is the “best” walking cycle for person representation? The key to this question is to mathematically model the motion information of a walking cycle, for which we adopt the sinusoid function, based on two observations: (1) Many bipedal robots use an intuitive walking method with sinusoidal foot [76]. It is based on the hypothesis that the trajectory of the feet follows sine waves in the *x*, *y* and *z* directions. That is consistent with the biomechanics of gait [77]. (2) The motion information of feet (horizontal positions) annotated on the iLIDS-VID dataset is almost exactly sinusoidal, as shown in Figure 4. Therefore, we model the horizontal displacements of feet as a sinusoid.

By modeling the horizontal displacements of feet as a sinusoid, we propose an effective criterion to select the “best” walking cycle. We expect the criterion has two characteristics: (1) it should be a complete walking cycle (i.e., a sinusoidal cycle) since it covers the entire dynamic information and variety of poses and shapes, and (2) it should be quite similar to sinusoid, namely, with less noise caused by cluttered background and occlusion. That is, we search the complete candidate walking cycles from the curves of the horizontal displacements according to the prior of walking persons. Then we evaluate how good a candidate walking cycle is, by measuring its fit error to a sinusoid.

**Figure 5:**
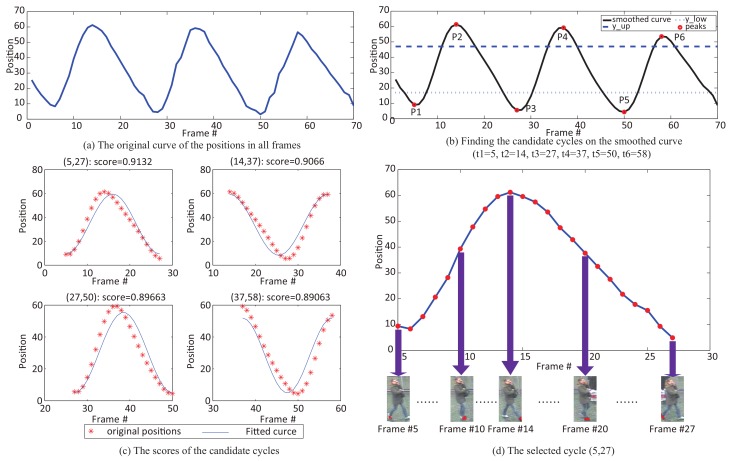
Walking Cycle Extraction. (**a**) the motion curve of a superpixel; (**b**) the smoothed curve, and the extreme points indicated as the red dot. (**c**) Four candidate cycles, (5,27), (14,37), (27,50), and (37,58), and their scores. (**d**) the final selected walking cycle (5,27).

Specifically, we first try to find candidate walking cycles from the motion information curves based on extreme points, as shown in Figure 5. Ideally, an extreme point corresponds to the postures when the distance of two legs is maximum. However, in the presence of noise and occlusions in practice, an extreme point with a small distance to the horizontal center line might be a false alarm. To obtain more accurate walking cycles, we process it in two ways: (1) we smooth the curve to extract more accurate extreme points by the least-squares polynomial fitting, and (2) we set upper bound y_up and lower bound y_low to eliminate the false alarms. The second way is based on the observation of the public datasets: the walking person is roughly cropped out in each frame, and approximately at the center of the frame. That means the horizontal center line is the symmetrical axis of two legs in a frame. Thus, we set the upper bound y_up and lower bound y_up with the same distance to the horizontal center line:

(2) $$\left\{\begin{array}{c} \begin{array}{cc} y\_up\; & =c+\lambda \end{array}\\ \begin{array}{cc} y\_low & =c-\lambda \end{array} \end{array}\right.$$

where λ is the threshold distance to the horizontal center line, *c* is the location of the horizontal center line, i.e., c=W/2, *W* is the width of the image. We set the bounds to filter the extreme points on the smooth curve, and alleviate the influence of noise and occlusions.

We denote by $\left(P_{1},P_{2},\ldots ,P_{K}\right)$ the *K* extreme points and by $t_{k}$ the frame number of the *k*-th extreme point $P_{k}$. Then we define a candidate walking cycle $\left(t_{start},t_{end}\right)$ based on a group of three consecutive extreme points $\left(P_{k},P_{k+1},P_{k+2}\right)$. If all three of them are bigger than y_up or smaller than y_low, this group will be considered as a candidate cycle ($t_{start}=t_{k}$, $t_{end}=t_{k+2}$).

To evaluate how “good” a candidate cycle $\left(t_{start},t_{end}\right)$ of *j*th superpixel is, we fit its positions ${\left\{L_{t}^{j}\right\}}_{t=t_{start},\ldots ,t_{end}}$ to sinusoid ${\left\{Q_{t}^{j}\right\}}_{t=t_{start},\ldots ,t_{end}}$ in a least-squares sense, and calculate the score $R^{j}\left(t_{start},t_{end}\right)$ with its fit error to sinusoid by:

(3) $$R\left(t_{start},t_{end}\right)=log\left(1-\frac{\sum_{t=t_{start},\ldots ,t_{end}}{\left|L_{t}^{j}-Q_{t}^{j}\right|}_{2}^{2}}{(t_{end}-t_{start}+1)*W}\right)$$

where *W* is the width of the image. The final selected walking cycle $\left(t_{start}^{*},t_{end}^{*}\right)$ is the one with the highest score of all the candidate cycles on the motion curves of all superpixels:

(4) $$\left(t_{start}^{*},t_{end}^{*}\right)=\underset{\left(t_{start},t_{end}\right)}{\mathrm{argmax}}R(t_{start},t_{end}).$$

We also present an example to visually illustrate our algorithm in Figure 5, corresponding to the scenario in Figure 2. For the *j*th superpixel $\left\{S_{1}^{j}\right\}$ shown in Figure 2b, we first smooth its motion information curve (as shown in Figure 5a), the smoothed curve is shown in Figure 5b. Figure 5b also indicates the extreme points with red dot. Next we evaluate these curves, the scores are calculated using their fit error to sinusoid, as shown in Figure 5c. The best cycle is selected with the highest score, as shown in Figure 5d. Note that, it may still suffer from background clutter and occlusions, although the proposed superpixel based method is more robust than pixel based methods. And selecting the best walking cycle can avoid this problem to some extent. Two more examples and more detailed discussion are shown and discussed in Section 4.3.

### 3.3. Superpixel-Based Representation

As mentioned in Section 1, a temporally aligned representation is proposed for video-based re-identification. In this paper, we refer to local maximal occurrence representation (LOMO) [8] to represent the individual frames. However, to further improve the robustness, we enhance the original LOMO in two aspects: (1) Fixed-sized patches are used in original LOMO. Although it is more robust to noise than aggregating pixel-level information, patches can span multiple distinct image regions, which can degrade the robustness. It is known that superpixels are superior to patches in many tasks because they can be considered as semantic visual primitives by aggregating visually homogeneous pixels. Thus, we propose to use superpixel-based LOMO to describe the still image. (2) The inclusion of background is another factor which may degrade the robustness of representation. To address this problem, person segmentation is employed to extract the masks of persons. And only the superpixels on the masks of the persons are considered for still image representation.

**Figure 6:**
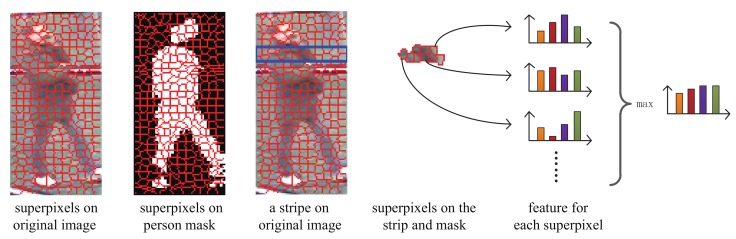
Illustration of the superpixel based representation.

To sum up, we proposed a superpixel-based LOMO (SPLOMO) representation for still images, as shown in Figure 6. Particularly, we first perform superpixel segmentation and person segmentation, using SLIC method [75] and Deep Decompositional Network (DDN) [78] respectively. Note that, the person mask is a binary map, where the semantic information of different parts is not used. Then for each strip, only the superpixels lie in this strip and the person mask are considered to compute the histogram based representation. In our implementation, for a given strip, a superpixel is considered when meeting two conditions: (1) its overlap with the corresponding person mask *O* is bigger than the threshold $T_{O}=0.8$; (2) its center is in the strip. The final feature is obtained by a max operation as in [8].

### 3.4. Temporally Aligned Representation

Given the selected walking cycle $\left(t_{start}^{*},t_{end}^{*}\right)$, the next problem for video-based re-identification is how to represent the 3D spatio–temporal video data and learn to match various person videos. This paper focuses on the former one. We propose a representation of a walking cycle, by temporally aligned pooling the descriptors of all the individual frames (or key frames) in the walking cycle. For the still images, we represent them using SPLOMO, introduced in Section 3.3.

**Figure 7:**
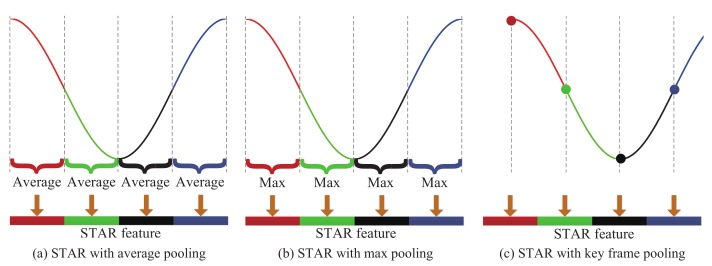
Illumination of temporally aligned pooling representation, with M=4. Three pooling manners for temporal alignment are presented: (**a**) average pooling, (**b**) max pooling, and (**c**) key frame pooling.

Note that the frame numbers of walk cycles in different video sequences are usually different, which is not convenient to learn a metric. An alternative modality is multi-versus-multi (MvsM), in which there is a group of multiple exemplars for each person in the gallery and group of multiple images of each person in the probe set. However, temporal information of a video sequence is missing in the MvsM method, while it is quite important in video-based re-id. To address this problem, we propose to use temporally aligned pooling method to normalize the representations of all the frames, according to the intrinsic periodicity property. That is, we perform temporally aligned pooling according to the sinusoid corresponding to the walking cycle. Specifically, we equally divide the sinusoid into *M* segments ${\left\{\Phi_{m}\right\}}_{m=1,\ldots ,M}$, and then describe the corresponding phases ${\left\{\Psi_{m}\right\}}_{m=1,\ldots ,M}$ to the *M* segments in the walking cycle. We describe the phase *m* as $F_{m}$ by temporally aligned pooling using the features of the images in $\Psi_{m}$. We finally concatenate ${\left\{F_{m}\right\}}_{m=1\ldots ,M}$ together to form a final representation, termed as superpixel-based temporally aligned representation (STAR). Three pooling manners are proposed for temporal alignment: average pooling, max pooling, and key frame pooling, as shown in Figure 7. It is worth noting that, $F_{m}$ is the feature of the first frame in the $\Psi_{m}$ in key frame pooling.

## 4. Experimental Results

In this section, we validate the proposed STAR method (The source code is available on https://github.com/chaunceygao/STAR) and compare it to other state-of-the-art approaches on three public video-based re-id datasets.

### 4.1. Datasets and Settings

#### 4.1.1. Datasets

**Figure 8:**
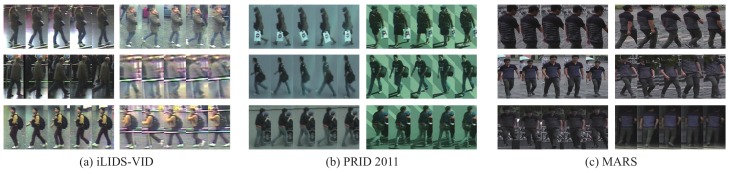
Example pairs of the image sequences of the same person in different camera views from two datasets. (**a**,**b**) shows images of one person with two different cameras in each row, in iLIDS-VID and the PRID 2011 dataset, (**c**) shows images of one person with six different cameras in MARS.

In this section, we conduct our experiments on three publicly available video datasets for video-based person re-id: the iLIDS-VID dataset [30], the PRID 2011 dataset [79] and MARS [49], as shown in Figure 8. The iLIDS-VID dataset consists of 600 image sequences for 300 people from two non-overlapping camera views, and each image sequence has variable length consisting of 23 to 192 image frames, with an average number of 73. The dataset is very challenging due to clothing similarities, cluttered background, occlusions, viewpoint variations across camera views (Figure 8a). The PRID 2011 dataset includes 400 images sequences for 200 people from two adjacent camera views. Each image sequence has variable length consisting of 5 to 675 image frames, with an average number of 100. In our experiments, the sequence pairs with more than 21 frames are used to the requirement on the sequence length for extracting walking cycles. The main challenges of the dataset are lighting and viewpoint variations across camera views (Figure 8b). The MARS dataset consists of 1261 identities captured by 2 to 6 cameras. The train and test sets contain 631 and 630 identities respectively. 20,175 tracklets are obtain by DPM detector [80] and GMMCP [81] tracker, among them 3248 are distractors due to false detection or tracking. A large number of tracklets contain 25–50 frames, and most pedestrians have 5–20 tracklets. The MARS dataset is more challenging due to distractors, detected or tracked bounding box, besides the challenges mentioned above (Figure 8c).

#### 4.1.2. Settings

In the proposed STAR method, SPLOMO is used to represent the still images and Cross-view Quadratic Discriminant Analysis (XQDA) [8] is used for metric learning. On iLIDS-VID and PRID 2011, the performance of all the methods is measured by the average Cumulative Matching Characteristics (CMC) curves after 10 trials. On MARS, the performance is evaluated by CMC with a fixed partition as [49], and by Mean Average Precision (mAP). Then, we give the parameters in our implementation. The width and height of the images in both datasets are W=64 and H=128 respectively. We perform superpixel segmentation using SLIC [75], with the maximal number of superpixel is 100, in both motion information extraction and superpixel based representation. The threshold distance to the horizontal center line λ=17, according to the datasets. We utilize average pooling manner for temporal pooling and divide a walking cycle into M=8 segments in our STAR method, according to the analysis in Section 4.4.

**Figure 9:**
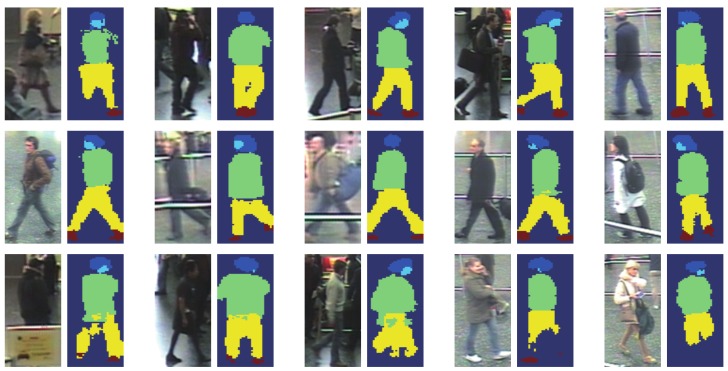
Some examples of person segmentation on the iLIDS-VID dataset.

In SPLOMO, we extract person masks using DDN (The code is available on http://mmlab.ie.cuhk.edu.hk/projects/luoWTiccv2013DDN/index.html) [78]. Figure 9 shows some examples of person segmentation by DDN, the person segmentation result used in this paper indicates the person region (see more details of DDN in [78]).

### 4.2. Comparison with the State-of-the-Art Methods

In this section, we report the comparison results of STAR with the existing state-of-the-art video-based person re-id approaches on iLIDS-VID, PRID 2011 and MARS datasets. Three groups of the approaches are compared, as shown in Table 1, e.g., (1) traditional methods: GEI + RSVM [59], HOG3D + DVR [30], Color + LFDA [82], STFV3D + KISSME [31], CS-FAST3D + RMLLC [57], SRID [55], TDL [56]; (2) deep network based methods: RNN [70], CNN + XQDA + MQ [49], SPRNN [53], ASTPN [28], DSAN [52]; and (3) TAPR [32] is the preliminary version of our method. More specifically, GEI+RSVM [59] is a gait based approach, which is not specially designed for person re-id. HOG3D + DVR [30], Color + LFDA [82], STFV3D + KISSME [31], CS-FAST3D + RMLLC [57] focus on appearance based representations for video. SRID [55] formulates the re-id problem as a block sparse recovery problem. TDL [56] mainly focuses on metric learning under the top-push constraint. [49] uses CNN to represent each frame. RNN [70], SPRNN [53], ASTPN [28], and DSAN [52] are end-to-end deep architectures, by incorporating feature leaning and metric learning together.

Table 1 shows that the STAR approach outperforms the other methods in general, especially on the iLIDS-VID and MARS datasets. In particular, on the iLIDS-VID dataset, the proposed method performs significantly better than the other methods, even the deep learning based methods. Specifically, the rank-1 and rank-5 identification rates of our method are 5.5% and 4.1% over the second-best scores, respectively. On the PRID 2011 dataset, although the deep learning based methods perform better, STAR obtains the comparative results and significantly outperforms the traditional methods. On the MARS dataset, although SPRNN [53] obtains the highest scores of rank-5 and rank-20, we achieve the comparable results. Moreover, the rank-1 score of STAR is 6.5% over the second-best score obtained by DSAN [52]. More importantly, the mAP of STAR is 70.0%, about 20% higher over SPRNN [53]. The comparison results on the three datasets demonstrate that the proposed method STAR performs favorably against the state-of-the-art methods, even deep learning-based ones. The comparison results of STAR with [30,31] show the superpixel level motion information is more robust than pixel-level motion information.

We also report the results of TAPR, which is the preliminary version of our method. The main difference of TAPR and STAR is that TAPR describes each frame with LOMO feature, while STAR describes it with a SPLOMO feature. As mentioned above, SPLOMO improves LOMO by introducing the constraint of superpixel and person masks for better spatial alignment. The comparison results of these two methods show that our proposed superpixel based representation improve the performance of LOMO distinctly, especially on the iLIDS-VID dataset. More specifically, the rank-1 identification rate is 67.5% for STAR on the iLIDS-VID dataset, while 55.0% for TAPR. This validates the effectiveness of the SPLOMO representation on spatial alignment.

It is worth noting that we achieve the best performance on both iLIDS-VID dataset and MARS dataset, and a comparable result on the PRID 2011 dataset. We believe this is because the iLIDS-VID and MARS datasets have more occlusions and cluttered background than the PRID 2011 dataset. And the proposed STAR can excavate the “best” walking cycle to reduce their effect and achieve more accurate temporal alignment. Moreover, STAR uses the person masks to avoid the effect of the background, and uses superpixel based representation to achieve better spatial alignment. This means (1) accurate spatial and temporal alignment is quite essential in the video-based re-identification, especially when the scenes are complex; (2) our method has great abilities in video temporal alignment even with complex scenes.

### 4.3. Examples of Selected Walking Cycles

**Figure 10:**
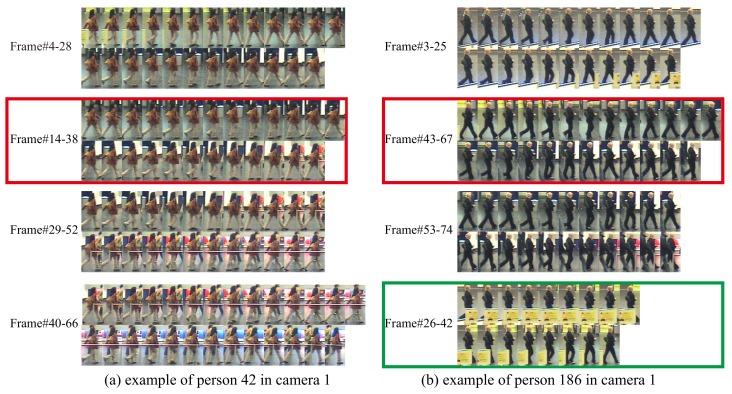
Two examples of selected walking cycles on iLIDS-VID. The candidate walking cycles for each video is presented, and the “best” walking cycle we selected is indicated by the red bounding box. Note that the image sequence in the green bounding box in (**b**) is not a candidate walking cycle. The frame indexes are given on the left side of the corresponding image sequences.

To alleviate the effect of occlusions and cluttered background, we propose to select the “best” walking cycle (a gait period). To further demonstrate the good performance of the proposed walking cycle selected method, we present two more examples in Figure 10 (we have given an example in Figure 5). All the candidate walking cycles are shown for each example, and the “best” walking cycle with highest score computed by Equation (3) is indicated by the red bounding box. From Figure 10a, we can find that the low portion of human in the first three images of the walking cycle *Frame#4-28* is partially occluded, and the walking cycles *Frame#29-52* and *Frame#40-66* both have cluttered background, while the walking cycle *Frame#14-38*, which is the selected one, has less noise than others. In Figure 10b, the walking cycle *Frame#3-25* suffers from occlusion, and the walking cycle *Frame#3-25* suffers from occlusion and clutter background, while the “best” one *Frame#43-67* has less noise. These examples demonstrate that our walking cycle method can select the ones with fewer occlusions and cluttered background. We believe that building a representation based on the selected walking cycle can alleviate the effect of occlusions and cluttered background.

Note that the image sequence *Frame#26-42* with the green bounding box in Figure 10b is not a candidate walking cycle. Since the low portion of a human in this sequence is heavily occluded, it is quite difficult to extract accurate motion information. While we can also observe that all the candidate walking cycles are complete gait periods. This validates the good performance of our motion information extraction method. Combining walking cycle selection and motion information extraction, the proposed method obtains an accurate walking cycle with fewer occlusions and cluttered background, which leads to accurate temporal alignment and robust representation.

### 4.4. Ablation Studies

#### 4.4.1. Evaluation of Temporally Aligned Pooling Manners

We evaluate the effects of the mentioned three temporally aligned pooling manners of our STAR algorithm on the iLIDS-VID dataset, i.e., average pooling (STAR_avg), max pooling (STAR_max), and key frame pooling (STAR_key), as shown in Table 2. The results show that the performance of STAR with average pooling is slightly better than that with max pooling and much better than that with key frame pooling. STAR_key performs the worst, for which we believe the reason is that it is very difficult to exactly localize the key points, due to discrete frames and noise. To demonstrate the effects of the temporal pooling manner, STAR with no pooling (STAR_no) is also reported in Table 2. We observe that STAR_no performs worse than STAR with a temporal pooling manner, even with key frame pooling. This validates the important role of temporally aligned pooling in video-based representation for re-id, and average pooling or max pooling is a good choice. STAR_avg performs best, thus we employ average pooling manner for quantitative comparison in Section 4.2.

Surprisingly, compared with Table 1, we observe that the proposed STAR with key frame pooling can perform comparably with TDL, and even STAR without pooling can outperform GEI + RSVM [59], HOG3D + DVR [30], Color + LFDA [82], STFV3D + KISSME [31], CS-FAST3D + RMLLC [57], and SRID [55]. This demonstrate the outstanding performance of the proposed method.

#### 4.4.2. Influence of Parameters

To devise the temporally aligned representations, we equally divide each walking cycle into *N* segments according to the corresponding sinusoid curve. Here we evaluate the effects of the number of segments *N*, as reported in Table 3. The results show that STAR with N=8 obtains the best performance, thus we divide a walking cycle into 8 segments for quantitative comparison in Section 4.2.

We also evaluate the influence of the number of superpixels in Table 4, which is set as 100 in our previous experiments. Note that we perform superpixel segmentation in both motion information extraction and image representation. The results show that STAR with 100 superpixels obtains the best performance, thus we set it as 100 in our experiments.

## 5. Conclusions

We have proposed a novel superpixel-based temporally aligned representation for video-based person re-identification. This representation focuses on both spatial and temporal alignment problems in video-based representations. To achieve temporal alignment, we select a video fragment of a walking cycle and describe the video fragment using temporally aligned pooling. To further improve spatial alignment, a superpixel is introduced to extract motion information and describe a still image. Unlike most previous video-based representations for re-id that use all the frames to build a spatio–temporal feature, we proposed to use only a “best” walking cycle, to reduce redundant information and simultaneously keep the “best” information. The extensive experiments, conducted on iLIDS-VID, PRID 2011 and MARS datasets, demonstrate that our method outperforms the state-of-the-art approaches.

## Figures and Tables

**Table 1:** Quantitative comparison of the proposed method and the state of the art methods on iLIDS-VID, PRID 2011 and MARS datasets. Bold and underlined values indicate the best and the second-best performance respectively.

Dataset	iLIDS-VID			PRID 2011			MARS			
Rank *R*	R=1	R=5	R=20	R=1	R=5	R=20	R=1	R=5	R=20	**mAP**
GEI + RSVM [59]	2.8	13.1	34.5	-	-	-	-	-	-	-
HOG3D + DVR [30]	23.3	42.4	68.4	28.9	55.3	82.8	-	-	-	-
Color + LFDA [82]	28.0	55.3	88.0	43.0	73.1	90.3	-	-	-	-
STFV3D + KISSME [31]	44.3	71.7	91.7	64.1	87.3	92.0	-	-	-	-
CS-FAST3D + RMLLC [57]	28.4	54.7	78.1	31.2	60.3	88.6	-	-	-	-
SRID [55]	24.9	44.5	66.2	35.1	59.4	79.7	-	-	-	-
TDL [56]	56.3	87.6	98.3	56.7	80.0	93.6	-	-	-	-
RNN [70]	58	84	96	70	90	97	-	-	-	-
CNN + XQDA + MQ [49]	53.0	81.4	95.1	77.3	93.5	99.3	68.3	82.6	89.4	49.3
SPRNN [53]	55.2	86.5	97.0	**79.4**	94.4	99.3	70.6	**90.0**	**97.6**	50.7
ASTPN [28]	62.0	86.0	94.0	77.0	95.0	99.0	44.0	70.0	81.0	-
DSAN [52]	61.9	86.8	98.6	77.0	**96.4**	**99.4**	73.5	85.0	97.5	-
TAPR [32]	55.0	87.5	97.2	68.6	94.6	98.9	-	-	-	-
STAR	**67.5**	**91.7**	**98.8**	69.2	94.9	99.1	**80.0**	89.3	95.1	**70.0**

**Table 2:** Evaluation of three different pooling manners on iLIDS-VID. Bold values indicate the best performance.

Methods	R=1	R=5	R=10	R=20
STAR_avg	**67.5**	**91.7**	**95.9**	**98.8**
STAR_max	65.6	91.3	96.1	98.7
STAR_key	56.2	87.4	94.8	98.3
STAR_no	52.8	83.5	90.8	95.7

**Table 3:** Evaluation of the number of segments in a walking cycle on iLIDS-VID. “N” in “STAR_N” is the number of segments. Bold values indicate the best performance.

Methods	*R* = 1	*R* = 5	*R* = 10	*R* = 20
STAR_1	52.8	83.5	90.8	95.7
STAR_2	64.7	88.9	93.8	97.9
STAR_4	67.3	90.8	95.5	98.4
STAR_8	**67.5**	**91.7**	**95.9**	**98.8**
STAR_16	66.2	**91.7**	95.7	98.6
STAR_32	65.9	91.5	95.8	98.7

**Table 4:** Evaluation of the number of superpixel on iLIDS-VID. Bold values indicate the best performance.

Superpixel Number	*R* = 1	*R* = 5	*R* = 20
50	65.9	88.0	99.0
75	65.9	89.4	**98.8**
100	**67.5**	**91.7**	**98.8**
125	66.7	90.0	98.7
150	65.8	88.6	98.7
